# The Philosophy of the Homœopathic Law of Cure, and the Advantage to the Dentist of a Correct Knowledge of Its Application

**Published:** 1892-11

**Authors:** Charles H. Taft

**Affiliations:** Cambridge, Mass.


### THE PHILOSOPHY OF THE HOMŒOPATHIC LAW OF CURE, AND THE ADVANTAGE TO THE DENTIST OF A CORRECT KNOWLEDGE OF ITS APPLICATION.[1]

BY CHARLES H. TAFT, D.M.D., CAMBRIDGE, MASS.

THE subject which I have chosen for my theme is one upon which nothing, to my knowledge, has ever appeared in print in the literature of our profession. In view of the rapid strides which dentistry has made within the last half-century, the character of the men who, with the wisdom born of a combined intelligence and rare inventive turn of mind, have brought the standing of our calling up to the full dignity of a liberal profession, the earnestness and the willingness of our brethren everywhere not only to seek after but to recognize truth wherever found, whatever its nature, it may seem strange that the grand principles of homœopathy should not long ere this have been recognized as furnishing one of the most important and efficient aids for the full and proper performance of all that the public expects of us as dentists. And yet, in view of the fact that in no dental school in the country, so far as I know, with one possible exception, is the materia medica taught on lines other than in harmony with the views of the allopathic or so-called regular school of medicine, it is not to be wondered at that when surgical or mechanical treatment fails to give the relief which the dentist often justly feels should follow the performance of an operation, he is then absolutely "at sea" as to what further course to pursue, unless it be to fall back upon palliatives, counter-irritants, and narcotics for the temporary relief of pain.

Before going further, let me assure my hearers that nothing is further from my purpose in this paper than to cast any discredit or reflection upon any school of medicine, or upon any one whose views in any way differ from mine. If I appear to be too aggressive, be assured it is merely because of my desire not only to disprove the assertion so often heard made, that medicine is not an exact science and never can be, but to prove that when practised in accordance with well-established laws it cannot possibly be anything else.

There exist to-day throughout the civilized world two promi-

---

[1] Read before the Massachusetts Dental Society, at its Twenty-seventh Annual Meeting, held at Boston, July 7, 1892.

nent schools of medicine: the one, the old, or so-called regular school of medicine; the other, the homœopathic, or new school of medicine. The former rests upon a mass of theories, experiments, and facts,—all good in themselves, to be sure, but without any effort or attempt being made to detach and put them together in their logical order or relation to each other. Without systematic organization of knowledge there can be no such thing as science, and since our friends of the old school make no claim that their system is founded upon any knowledge other than what is purely empirical, I believe they are honest when they affirm that medicine is not a science and never can be, and I agree with them fully so far as their method of practising it is concerned. Nor is it to be wondered at that without a well-defined knowledge of the toxical and medicinal action of the various drugs which the physician may make use of, and in the absence of any good and sufficient reason or principle whereby he chooses a drug, or more generally mixes several of them together for the treatment of disease, he might naturally mistrust the foundation upon which he stands in the practice of his profession, as well as his ability to effect a speedy relief from pain or a permanent cure.

How different is the case with him who is guided in his methods and treatment of disease by principles founded upon purely inductive and deductive processes of reasoning, leading up to a well-established law of cure as scientific and universal in its application as the laws governing the movements of the heavenly bodies, and indeed all the natural or physical laws which govern the whole universe!

The homœopathist claims that medicine, when practised in accordance with the teachings of Hahnemann, is therefore not only a science but an exact science, because it rests upon a fundamental law; and we come now, first, to the consideration of this law of cure, and afterwards to the consideration of how it concerns the dentist in his every-day practice.

Briefly stated, this law is expressed by the phrase *Similia similibus curantur*, which means that for the proper treatment or cure of any disease a medicine must be selected which would produce symptoms similar to those manifested when administered to a person in health.

To test the correctness of this law one has only to prove it upon himself or others to be convinced of its accuracy.

The first question, therefore, which naturally arises when a patient presents himself with any particular disturbance of the vital force is, What medicine will produce a similar group of symptoms when taken in a state of health? When, after carefully making note of all the symptoms, subjective as well as objective, we select a remedy which reaches the totality of all the symptoms,—in other words, when we have found the similimum, or similar remedy,—we give the medicine with the utmost confidence that it will produce the desired result. There is no guess-work nor any hit-or-miss principle about it, as obtains where the physician is guided by no scientific principle in the selection of his remedies.

We recognize, therefore, that every medicine must have a certain definite action; first, its physiological or toxical effect upon the different organs and tissues of the body, and, secondly, its medicinal or curative action. By studying the physiological action of a given drug we find that it has certain characteristics which distinguish it from other drugs. We find that different drugs affect in peculiar and in different ways the brain, the heart, the lungs, the digestive organs, the bones, the muscles, the mucous membranes,—in fact, all the different organs and tissues of the body,—each one producing well-defined symptoms in localized parts of the system, and by the process of reflex action affecting thereby the whole system.

Conversely, by studying the nature of disease, we recognize that there is, as Hahnemann says, a disturbance of the vital force which manifests itself in plainly-marked symptoms. In other words, the organs or tissues, which are for the time being in a condition of abnormal functional activity, find themselves not in harmony with the rest of the system, and to effect a proper restoration to health of the parts concerned, harmony must be restored.

To demonstrate the philosophy of the homœopathic law, therefore, we must show that the only intelligent and scientific method of restoring harmony is by the administering of what is termed the similimum or similar remedy, and this brings us naturally to the consideration and proper understanding of the laws which govern the action of drugs: the first law being, that every drug produces two entirely opposite effects: the first, the primary and transient effect; the second, the secondary and permanent effect.

To make this law clear by illustration: an old-school physician prescribes, for instance, a cathartic for constipation, and what is the result? The patient soon has a violent diarrhœa, the intestines being forced to an unusual and an unnatural activity. In a short time the primary effect passes away and the secondary effect takes its place, leaving the intestines again in a state of torpor and the same or a more aggravated condition than before. The relief has been, to be sure, a temporary one, but the subsequent condition is worse than the first.

Again, morphine is a drug too often employed for the relief of pain and to simulate sleep. Its primary effect accomplishes this, but what is its after-effect? Increased sensibility and restlessness, which requires in every case an increased dose to again allay. Many other examples might be cited, did time permit.

The second and equally important law is, that precisely opposite effects can be produced by the same drug, according to the quantity in which it is administered. The idea has long prevailed in the old school that if a drug was good for anything it must be administered in a large dose to be productive of any good; but this idea, fortunately for humanity, is fast falling into a state of innocuous desuetude, and the practice of allopathic physicians in this respect is becoming very materially modified. The remark of Dr. Oliver Wendell Holmes in this connection, and his scepticism as to the value or efficacy of medicines when so administered, so far as their therapeutic value is concerned, is too familiar to us all to call for any repetition here on my part.

To illustrate this second law: we know that small doses of rhubarb and calomel sometimes allay irritation of the bowels and cure diarrhœa and dysentery, while large doses produce precisely opposite effects. Again, small doses of ipecac and tartar emetic may be given for their curative action in cases of disordered stomach with vomiting, etc., while large doses of the same medicines bring about these same abnormal conditions. Small doses of cinchona may cure chills and fever, while large doses produce the same conditions; and so on, in countless similar ways, do we see the action of drugs operating for good or evil when administered in sickness or in health, in accordance with well-established laws.

How simple, then, and easy of comprehension is the homœopathic law of cure, and with what force does it commend itself to our reason and intelligence! The difficulty which seems to present itself most strongly to the opponents of homœopathy is the inability or rather, perhaps, the unwillingness to recognize that there can be any power or force in anything not plainly presented to our bodily senses, and in what may honestly appear to them to be contrary to ordinary common sense, and so rather than helping by patient investigation and study to add to the sum of human knowledge and human happiness, so far as their desire of arriving at any purely scientific system of medicine is concerned, they are contented to stand aside, and make general assertions and denials, which, being expressions of opinion only, have no scientific value, and are therefore worthless.

But it is no new thing for truth of any kind to meet with abundant opposition. The reception of the discoveries which followed the invention of the telescope, and the ridicule and condemnation to which Galileo was subjected for the promulgation of his theories as to the laws governing the movement of the heavenly bodies, is only equalled by the same ridicule and opposition which, even in this enlightened age, is meted out with a liberal hand not only upon a now universally recognized system of medicine, but upon all who have the courage to practise medicine in accordance with its teachings. But let the doubter in our own profession, and all others who take exceptions to the truth of the homœopathic law of cure, do as I have done,—put the principle of it into every-day practice. I can assure you the results of your work, when once you begin to thoroughly understand your subject, will surprise and astonish you. Lest a man may not have sufficient faith in the efficacy of the higher potencies, I would advise him to first begin with the lower ones, discarding them as his success and familiarity with them lead him to a greater and more satisfactory success with the higher ones.

We come now to the second part of our subject, and I can, perhaps, best illustrate the method of applying the homœopathic law by citing a few cases in practice.

Suppose, for example, a patient presents himself suffering with severe pain in the teeth or face, the result, it may be, of an exposed pulp, an inflammation of the pericementum, a gathering abscess, or the result of other causes producing an annoying train of symptoms. Our first aim is to select the proper remedy which will afford the speediest relief, and in order to make the right selection we must first compare the symptoms of the patient with the remedies which apply to them, and then compare the medicines selected with the symptoms in question.

It is my practice to take careful note of every symptom, writing them down if necessary, getting, so to speak, a pen-picture of the case. Among the various symptoms one may readily recognize one or more key-note symptoms, which instantly suggest a given remedy. It may happen that not all of the symptoms of your patient will be found under the drug which seems to be the indicated one, in which case it will be necessary to turn to other drugs and compare the symptoms as laid down under those remedies. But it must so happen that all, or at least the greater part, must be found under the drug selected, in order to give the desired relief or to effect a permanent cure; and when such a selection is made, you give the medicine to your patient with the utmost confidence and assurance that it will produce the desired result. There is no guess-work nor humbug about it, and your patient will not be likely to attribute the curative action of the medicine to his imagination, or be so ungenerous as to say that relief would have come at just that time without the medicine.

To make my meaning still clearer, permit me to illustrate by one or two cases in practice:

On February 8, 1892, Miss W. came to my office with a large corono-distal cavity in the right inferior second bicuspid. Careful excavating failed to avoid an exposure of the pulp, which was carefully treated, and capped by applying oil of cloves, and dusting a thin layer of the dry oxide of zinc directly over it, then filling the cavity with oxyphosphate, avoiding all pressure. This, I may say in passing, was a case which, when treated in this manner, rarely gives my patients any further trouble. But this one proved an exception to the general rule. The patient began to suffer almost immediate discomfort, which grew worse from day to day, and continued with almost uninterrupted pain for nearly three weeks. The patient was as unwilling to have the pulp destroyed as I was to suggest its being done as a *dernier ressort*, and temporary relief was afforded from day to day with capsicum plasters until they were without further efficacy. On February 27 she came to my office almost frantic with pain, and saying she could bear it no longer. The following symptoms were present: Great soreness and lameness of the tooth; soreness of the gums; pains extending into the upper teeth and round to the wisdom tooth on the left side. Aggravated in the cold air and by hot and cold things taken into the mouth; worse after eating and in the early part of the evening and night; relieved by hot external applications; great dulness and tired feeling in the eyes.

Turning to the repertory, we find the following remedies for—

1. Soreness and lameness of the teeth: Arsenicum, belladonna, bryonia, causticum, mercurius, nux vomica, sulphur.

2. Soreness of the gums: Bryonia, carbo vegetabilis, graphites, mercurius, natrum muriaticum, rhus, pulsatilla.

3. Pains extending to upper teeth: Nux vomica, sulphur, rhus tox., hyoscyamus, lachesis, mercurius.

4. Worse in cold air: Hyoscyamus, belladonna, mercurius, calcarea carb., staphisagria, sulphur.

5. Hot and cold things in the mouth: Rhus, bryonia, calcarea, nux, pulsatilla, staphisagria, sulphur, mercurius.

6. Worse in early part of the evening: Bryonia, chamomilla, natrum muriaticum, rhus, sulphur.

7. Relief from hot applications: Arsenicum, belladonna, calcarea, hyoscyamus, chamomilla, lachesis, mercurius, nux vom., pulsatilla, rhus, staphisagria, sulphur.

8. Dulness of eyes: Arsenicum, belladonna, bryonia, hyoscyamus, mercurius, nitric acid.

After striking out all the remedies which occur only once or twice, we find that arsenicum appears three times; natrum muriaticum, four times; pulsatilla, three times; staphisagria, four times; hyoscyamus, four times; belladonna, five times; nux vomica, five times; rhus, five times; bryonia, six times; sulphur, seven times; mercurius, eight times.

If we now examine the symptoms in the materia medica under mercurius and sulphur, we shall find that these will be the indicated remedies, and we give the preference to mercurius, as that more nearly reaches the totality of all the symptoms.

This remedy was, therefore, given in the above case in the two-hundredth potency, with absolute confidence that it would quickly give the desired relief. I neither saw nor heard from the patient for a month, when I received a letter, stating that "the medicine had served its purpose well, that the tooth had given her no further discomfort, and that she felt she should have no further trouble with it, and was furthermore heartily glad that the pulp had not been destroyed."

Frequently a patient presents himself, complaining of severe pain in a tooth, which, we have reason to suspect, may be abscessed, although the process of suppuration has not become sufficiently advanced to cause any lameness or soreness, either in the tooth or peridental membrane. It may happen that the tooth has a large filling, and from the appearance we may expect the pulp has died beneath the filling, but we do not wish to remove it only to find possibly a live pulp. No fistulous opening exists; the teeth and tissues about the affected region all present a healthy appearance, and the fillings are all in good order. The patient, nevertheless, is suffering violent pain, which demands immediate relief. What is to be done in such a case?

My own practice would be such as was adopted in the case of Mr. S., a student at Harvard, who came to my office on May 3, complaining of severe pain in the left inferior first molar with the following symptoms: Pain commenced the previous afternoon, growing worse towards evening, and kept him awake nearly all night. No pain nor soreness in the tooth itself or gums, but a dull, steady ache, starting in that tooth, and radiating through the bones of the face and up into the temple. Aggravated by hot things and after eating; also worse in the warm room. Cold water held in the mouth gave relief, but only temporarily; also relieved by being out in the open air. Could eat on that side with perfect comfort. Patient remarked that the tooth (the sixth-year molar, bear in mind) was dead and had been previously treated. His symptoms, it seemed to me, pointed strongly to bryonia, which was accordingly given in the divided dose,—that is to say, dissolved in water, one tablespoonful every half-hour until relieved. He came back in two or three hours, reporting no relief whatever.

Knowing, therefore, that I had not selected the right remedy, I made the case the subject of still further study. Noticing a large amalgam filling in the crown of the twelfth-year molar back of it, and suspecting a gathering abscess at its root, although there was no soreness in the tooth whatever, and nothing to indicate any possible cause of the trouble except the filling, I prescribed silica to confirm my diagnosis and to hasten the process of suppuration, saying to the patient that the medicine would in all probability give him speedy relief, and that he would come in to see me the following day with his face considerably swollen. He appeared at my office the following afternoon, reporting entire relief from pain within two hours after he left me the day before, when he went to sleep and slept all night, awakening in the morning to find his face in the swollen condition I had anticipated.

Examination showed at once that the twelfth-year molar and not the sixth was the offending tooth, which was then at once opened into and treated.

The above are cited as illustrations of the many cases that come up in the daily practice of every dentist, and were it within the limits of the paper, I should be only too happy to give many more to show how easily and quickly all sorts of disturbances of the vital force affecting the oral cavity, which we as dentists are expected to know how to intelligently treat, may be made to yield to the gentle curative action effected by the high potencies of the proper indicated remedies.

It not infrequently happens that a patient, unaccustomed to homœopathic treatment, will express an incredulity and great scepticism as to the power of the innocent-looking medicine to do all that you assure him it will do; but I have yet to find such a patient who not only gratefully but generously gives the credit of his relief from suffering to the action of the medicine, and who is not at the same time the most enthusiastic sort of an individual, simply because he cannot understand *how* the medicine should work as it does. *That* it does he is amply convinced. Of the fact that it was not his imagination, he is also convinced.

In conclusion, let me say that if I have succeeded in awakening any interest in a subject intensely interesting to me, as I bring the application of it more and more into a busy every-day practice, I shall not regret having so heavily taxed your time and patience at this somewhat late hour. When once you begin your personal investigation, and study to become thoroughly acquainted with the action of drugs and the laws which govern it, and have tested by actual observation and experience the much greater efficacy of the higher potencies over the lower ones in some of the commonest kinds of toothache even, you will then, at least, have given yourself the satisfaction of rendering to your patients some real benefit, as well as having begun the study of the art of healing upon accurate, and therefore necessarily scientific, principles.

